# Safety and Efficacy of Right‐ Versus Left‐Sided Transvenous Lead Extraction

**DOI:** 10.1111/jce.70497

**Published:** 2026-09-19

**Authors:** Nadine Molitor, Caroline Wiederkehr, Jan Steffel, Andreas Häberlin, Alexander Breitenstein, Daniel Hofer

**Affiliations:** ^1^ Division of Electrophysiology, Department of Cardiology, University Heart Center University Hospital Zurich Zurich Switzerland; ^2^ Department of Cardiology, Inselspital, Bern University Hospital University of Bern Bern Switzerland; ^3^ ARTORG Center for Biomedical Engineering Research, University of Bern Bern Switzerland

**Keywords:** complication, lead extraction, right, right‐sided, success

## Abstract

**Background and Aims:**

Transvenous lead extraction (TLE) is increasingly performed for malfunction or infection of cardiac implantable electronic devices. Because of anatomic differences, TLE for right‐sided (RS) electrodes may differ in outcome. This study aimed to evaluate whether RS TLE differs in patient characteristics and outcomes compared to left‐sided TLE.

**Methods:**

We retrospectively analyzed patients who underwent TLE at the University Hospital Zurich from 2013 until 2023 regarding clinical and radiological success and complications.

**Results:**

A total of 556 patients underwent 568 TLE from January 2013 until December 2023. Patients with RS TLE procedures (*n* = 147, 25.9%) were older, more often PM‐dependent, had longer lead dwell time, more leads with passive fixation and were more often extracted for infection. RS TLE procedures had a longer procedure duration (130 vs. 108 min, *p* = 0.002), more often needed a snare‐bailout strategy (22% vs. 10%, *p* = 0.0.45), resulted in lower radiological success rates (88.4% vs. 93.8%, *p* = 0.045) and more major as well as minor complication rates.

**Conclusion:**

In our retrospective single‐center analysis, RS TLE Procedures were more challenging concerning patient characteristics and resulted in lower TLE success as well as higher complication rates.

**Abbreviations**
CAD: coronary artery disease
CIED: cardiac implantable electronic devices
DCM: dilated cardiomyopathy
HCM: hypertrophic cardiomyopathy
HOCM: hypertrophic obstructive cardiomyopathy
LS: left sided
LVEF: left ventricular ejection fraction
NCCM: non‐compaction cardiomyopathy
PFO: persistent foramen ovale
PM: pacemaker
RS: right sided
TLE: transvenous lead extraction

## 1 Introduction

Transvenous lead extraction (TLE) has become an integral component of contemporary management of cardiac implantable electronic devices (CIEDs), primarily driven by lead malfunction, device upgrades, and CIED‐related infections. Over the past decades, increasing implantation rates and longer patient survival have resulted in a growing population of patients requiring lead extraction, thereby emphasizing the need for a thorough understanding of procedural risks and outcomes [1, 2, 3].

Most available data on TLE are derived from left‐sided (LS) procedures, which represent the standard implantation approach. However, right‐sided (RS) lead implantation is performed in a relevant subset of patients due to venous occlusion, prior infections, congenital anatomical variants, or operator preference. Anatomical differences between the right and left venous systems, including variations in vessel angulation, lead course, and proximity to adjacent structures, may influence procedural complexity as well as success and complication rates of TLE.

Despite these potential differences, comparative data on outcomes of RS versus LS TLE remain limited. Therefore, the present study aimed to evaluate whether RS TLE differs from LS TLE with regard to patient characteristics, procedural success, and clinical outcomes.

## 2 Methods

### 2.1 Study Design

We conducted a retrospective analysis of all patients who underwent TLE at the University Hospital Zurich between January 2013—marking the initiation of a standardized and supervised extraction program—and December 2023. Each TLE procedure was analyzed as an independent event, irrespective of patient identity, in order to include multiple extractions of the same patient, provided that subsequent procedures were not performed during the same hospitalization. Patients who underwent lead explantation (see Table 1, *Definitions*) or lead extraction as part of an open‐chest surgical procedure were deliberately excluded. Due to the retrospective study design, the selection and sizing of extraction tools were at the discretion of the operating physician. Notably, only mechanical rotational lead extraction systems were utilized throughout the study period. The study protocol was reviewed and approved by the local ethics committee (BASEC‐Nr: 2018‐01540).

**Table 1:** Definitions.

Term	Definition
Lead explantation	Removal of a lead using simple traction techniques, with a lead dwelling time of <1 year.
Lead extraction (TLE)	Removal of at least one lead with a dwelling time exceeding 1 year.
Complete radiological success	Removal of all targeted leads and associated material, with no residual lead fragments remaining.
Partial radiological success	Presence of residual lead fragments <4 cm following TLE.
Radiological failure	Inability to remove the targeted lead(s), with >4 cm of lead remaining.
Clinical success	Achievement of the intended clinical outcome of the TLE procedure. This may occur even in cases of partial radiological success or radiological failure, provided that residual lead fragments do not increase the risk of adverse events (e.g., perforation, embolic events, persistence of infection) and in the absence of any procedure‐related major complications resulting in permanent disability or death.
Clinical failure	Failure to achieve the intended clinical outcome of TLE.
Major complications	Any complication causing persistent or significant disability, life‐threatening events, or death, or necessitating surgical intervention to prevent such outcomes.
Procedure‐related major complications	Complications directly attributable to TLE that require surgical intervention, prolong hospitalization, cause persistent or significant disability, are life‐threatening, or result in death.
Minor complications	Any adverse event occurring during the same hospital stay as TLE that requires medical intervention, observation, or minor procedural intervention without prolonging hospitalization or causing persistent/significant disability, life‐threatening events, or death.
Reoperation	Any surgical or interventional procedure performed during the same hospital stay to address a complication following TLE, whether procedure‐related or unrelated.

### 2.2 Data Collection and Analysis

Each patient case was individually evaluated to assess procedural success, failure, and complications for 30 days after TLE. Data were collected through a systematic review of operative reports, discharge summaries, and chest radiographs. All patient data were fully anonymized prior to analysis. Categorical variables are expressed as numbers and percentages. Continuous variables are presented as median and interquartile range (IQR). A Mann‐Whitney U test was used to compare procedural and outcome data. Proportions were compared using Fisher's exact test. Univariate and multivariate binary logistic regression models were fitted to quantify the association between selected baseline variables and the need for extraction sheath upsizing. Variables were selected a priori based on previously described factors, which may impede transvenous lead extraction. Moreover, procedure‐related variables were assessed. The full multivariate model included all variables from the univariate models with a *p*‐value < 0.1. An α‐level of ≤0.05 was considered significant. For statistical analysis, R version 4.1.1 for Windows (R Foundation, Vienna, Austria) was used.

### 2.3 Definitions

Definitions were aligned as closely as possible with the 2009 and 2017 HRS expert consensus statements and the 2018 EHRA expert consensus statement on lead extraction, as well as with definitions applied in large multicenter registries, to ensure comparability (Table 1). However, due to incomplete documentation within patient records, several modifications were necessary relative to these expert consensus statements and registries [1, 2, 3]. First, differentiation between intra‐procedural and post‐procedural complications was not feasible because of the substantial lack of temporal specification in the available data. Second, given that patients frequently underwent additional procedures during hospitalization, attribution of complications to a specific intervention was often uncertain. Therefore, all complications documented in the medical record during the hospital stay related to TLE were reported. Consistent with the EHRA and HRS expert consensus statements, clinical success of TLE was considered achievable even in the absence of radiological success, as the assessment of clinical outcome primarily depends on the indication for extraction. In cases of infection, only complete removal of the cardiac implantable electronic device (CIED) was regarded as clinical success, although a residual lead fragment of <4 cm may be acceptable according to the consensus [1]. For non‐infectious indications, clinical success could also be assumed despite a residual fragment >4 cm [1], provided that the remaining segment was not expected to increase the risk of adverse events (e.g., vascular obstruction) and in the absence of major procedure‐related complications. Mortality following TLE was assessed up to 30 days post‐procedure. The following section outlines the specific definitions applied in this study regarding the TLE procedure, procedural success and failure, as well as associated complications.

## 3 Results

A total of 556 patients underwent transvenous extraction in 568 procedures between January 2013 and December 2023. Procedures were performed by four operators, with a single operator responsible for 71.4% of all extractions, highlighting a high level of procedural consistency and operator experience.

Patients with right‐sided CIED implantation were significantly older than those with left‐sided implantation (median 69 vs. 66 years, *p* = 0.006) and were more frequently Pacemaker dependent (53.7% vs. 27.1%, *p* < 0.001). Underlying cardiac disease differed significantly between groups (*p* < 0.001). Right‐sided implantation was more commonly observed in patients with primary electrical heart disease, whereas coronary artery disease, dilated cardiomyopathy, and valvular heart disease were more prevalent among patients with left‐sided implantation. Indications for lead extraction also differed significantly (*p* = 0.014), with lead dysfunction being more frequent in the left‐sided group, while systemic infection and device upgrade were relatively more common in the right‐sided group. In addition, right‐sided implantation was associated with a longer dwell time of the oldest lead (median 11.9 vs. 7.1 years, *p* < 0.001), a significantly higher number of previous device implantations (*p* < 0.001), and a higher prevalence of passive lead fixation (50.3% vs. 18.1%, *p* < 0.001). Patients with left‐sided implantation had a significantly lower left ventricular ejection fraction (median 48% vs. 54%, *p* = 0.003) (Table 2).

**Table 2:** Baseline characteristics.

Baseline characteristics	Overall 568	CIED implanted on right side	CIED implanted on left side	*p*‐value
Age [years]	67 (56–76)	69 (60–77)	66 (55–75)	0.006
Female gender [*n*]	170 (46.4%)	49 (33.3%)	121 (28.1%)	0.30
Hypertension [*n*]	309 (54.4%)	86 (58.5%)	223 (53.0%)	0.25
Chronic kidney disease [*n*]	193 (34.0%)	45 (30.6%)	148 (35.2%)	0.36
Diabetes [*n*]	117 (20.6%)	28 (19.0%)	89 (21.1%)	0.64
PM‐dependent [*n*]	193 (34.0%)	79 (53.7%)	114 (27.1%)	<0.001
Lead dwell time (oldest lead) [years]	7.8 (4.4–12.4)	11.9 (5.8–18.5)	7.1 (4.1–10.4)	<0.001
Passive lead fixation [*n*]	150 (26.4%)	74 (50.3%)	76 (18.1%)	<0.001
LVEF [%]	50 (35–57)	54 (43–59)	48 (33–57)	0.003
PFO present in TEE [*n*]	62 (10.9%)	20 (13.6%)	42 (10.0%)	0.22
Right‐left shunt [*n*]	38 (6.7%)	12 (8.2%)	26 (6.2%)	0.44
Antiplatelet therapy				0.16
Single antiaggregation [*n*]	150 (26.4%)	30 (20.4%)	120 (28.5%)	
Dual antiaggregation [*n*]	11 (1.9%)	3 (2.0%)	8 (1.9%)	
Anticoagulation [*n*]	164 (46.5%)	72 (49.0%)	192 (45.6%)	0.50
Number of previous device interventions				<0.001
0	140 (24.6%)	31 (21.1%)	109 (25.9%)	
1–2	335 (59.0%)	75 (51.0%)	260 (61.8%)	
3–4	72 (12.7%)	30 (20.4%)	42 (10.0%)	
≥5	21 (3.7%)	11 (7.5%)	10 (2.4%)	
Underlying cardiac disease				<0.001
CAD [*n*]	133 (23.4%)	14 (9.5%)	119 (28.3%)	
DCM [*n*]	79 (13.9%)	6 (4.1%)	73 (17.3%)	
Valvular heart disease [*n*]	43 (7.6%)	14 (9.5%)	29 (6.9%)	
Primary el. heart disease [*n*]	247 (43.5%)	99 (67.3%)	148 (35.2%)	
Congenital heart disease [*n*]	12 (2.1%)	6 (4.1%)	6 (1.4%)	
HOCM [*n*]	6 (1.1%)	0 (0.0%)	6 (1.4%)	
HCM [*n*]	6 (1.1%)	2 (1.4%)	4 (1.0%)	
NCCM [*n*]	6 (1.1%)	0 (0.0%)	6 (1.4%)	
Other [*n*]	32 (5.6%)	6 (4.1%)	30 (7.1%)	
Indication for extraction				0.014
Local infection [*n*]	56 (9.9%)	20 (13.6%)	36 (8.6%)	
Systemic infection [*n*]	97 (17.1%)	32 (21.8%)	65 (15.4%)	
Lead dysfunction [*n*]	304 (53.5%)	61 (41.5%)	243 (57.7%)	
Chronic pain [*n*]	13 (2.3%)	4 (2.7%)	9 (2.1%)	
Upgrade [*n*]	72 (12.7%)	24 (16.3%)	48 (11.4%)	
Recalled lead [*n*]	2 (0.4%)	0 (0.0%)	2 (0.5%)	
Lack of indication/patient wish [*n*]	12 (2.1%)	1 (0.7%)	11 (2.6%)	
Other [*n*]	12 (2.1%)	9 (6.1%)	14 (3.3%)	

*Note:* Median values with interquartile ranges in brackets and numbers with percentages are shown.

Abbreviations: CAD, coronary artery disease; DCM, dilated cardiomyopathy; HCM, hypertrophic cardiomyopathy; HOCM, hypertrophic obstructive cardiomyopathy; LVEF, left ventricular ejection fraction; NCCM, non‐compaction cardiomyopathy; PFO, persistent foramen ovale.

### 3.1 Radiological and Clinical Success

Among the 568 TLE procedures, complete radiological success was achieved in 525 cases (92.4%), with an additional 22 procedures (3.9%) resulting in partial radiological success, yielding an overall combined radiological success rate of 96.3%. Radiological failure occurred in 21 procedures (3.7%). Clinical success was observed in 336 procedures (96.0%). Clinical failure occurred in 14 cases (4.0%), comprising 8 deaths within 30 days post‐procedure, 3 permanently disabling complications, and 3 instances of insufficient lead removal. Complete radiological success was achieved less frequently in right‐sided compared with left‐sided lead extractions (88.4% vs. 93.8%, *p* = 0.045), while partial radiological success was correspondingly more common (*p* = 0.045). Clinical success rates were high and did not differ significantly between groups (92.5% vs. 94.8%, *p* = 0.311). Procedure and fluoroscopy duration were significantly longer in patients undergoing extraction of right‐sided CIEDs (procedure median 130 vs. 108 min, *p* = 0.002, fluoroscopy median 13 vs. 8 min, *p* = 0.071). Femoral snare techniques were used more frequently in RS extractions (22.8% vs. 10.0%, *p* = 0.045) (Table 3).

**Table 3:** Procedural and outcome data.

	Total	CIED implanted on right side	CIED implanted on left side	*p*‐value
Duration of intervention [min]	117 (89–159)	130 (93–179)	108 (87–150)	0.002
Fluoroscopy duration [min]	8.7 (4.4–20)	13 (6–28)	8 (4–15)	0.071
General anesthesia [*n*]	483 (85.0%)	130 (88.4%)	353 (83.8%)	0.226
Extraction tools used				
None/simple traction [*n*]	46 (13.1%)	26 (17.7%)	65 (15.4%)	0.516
Mechanical non‐powered sheath [*n*]	28 (4.9%)	9 (6.1%)	19 (4.5%)	0.257
Rotational mechanical sheath [*n*]	343 (60.4%)	85 (57.8%)	258 (61.3%)	0.493
Spectranetics [*n*]	179 (31.0%)	51 (35.8%)	128 (31.8%)	0.354
Femoral snare [*n*]	65 (11.4%)	23 (22.0%)	42 (10.0%)	0.045
Use vegetation debulking system (Angiovac) [*n*]	13 (2.3%)	3 (2.0%)	10 (2.4%)	1
Use of cerebral protection device [*n*]	5 (0.9%)	3 (2.0%)	2 (0.5%)	0.112
Outcome				
Complete radiological success	525 (92.4%)	130 (88.4%)	395 (93.8%)	0.045
Partial radiological success	22 (3.9%)	10 (6.8%)	12 (2.9%)	0.045
Clinical success	535 (94.2%)	136 (92.5%)	399 (94.8%)	0.311
Complications				
Major complications	27 (4.8%)	13 (8.8%)	14 (3.3%)	0.012
Minor complications	93 (16.4%)	33 (22.4%)	60 (14.3%)	0.027
Re‐intervention due to complication	28 (4.9%)	13 (8.8%)	15 (3.6%)	0.015
Perioperative death	8 (1.4%)	1 (0.7%)	7 (1.7%)	0.687
Death within 30 d	17 (3.0%)	8 (5.4%)	9 (2.1%)	0.052

*Note:* Median values with interquartile ranges in brackets and numbers with percentages are shown.

### 3.2 Complications

Complications occurred in 120 of 568 TLE procedures (21.1%), of which 93 (77.5%) were classified as minor 27 (12.1%) as major. Reoperations due to complications were required in 27 cases (4.7%). A total of 27 major complications occurred in 19 TLE procedures, representing 4.8% of all procedures. A total of 82 minor complications occurred in 68 of 350 TLE procedures (19.4%). The most frequent minor complication was hematoma not requiring intervention (5.1%), followed by newly developed severe tricuspid regurgitation and arm swelling/thrombosis (each 2.9%), pericardial effusion not requiring intervention, and blood transfusion (each 2.3%), among other events. Major (8.8% vs. 3.3%, *p* = 0.012) and minor (22.4% vs. 14.3%, *p* = 0.027) complications occurred more frequently in the RS group. Re‐intervention due to complications was also more common after right‐sided extraction (8.8% vs. 3.6%, *p* = 0.015) (Table 4).

**Table 4:** Major and minor complications.

		Implanted on right side (*n* = 147)	Implanted on left side (*n* = 421)	*p*‐value
Major Complications	Death	6 (4.1%)	5 (1.2%)	0.039
	Perforation	2 (1.4%)	1 (0.2%)	0.165
	Vascular perforation	0	1 (0.2%)	1
	Hematothorax	1 (0.7%)	1 (0.2%)	0.45
	Cardiac decompensation	2 (1.4%)	3 (0.7%)	0.61
	Sepsis	1 (0.7%)	0	0.25
	Multiple organ failure	2 (1.4%)	1 (0.2%)	0.165
	Stroke	2 (1.4%)	1 (0.2%)	0.165
	Anesthesia complications requiring intervention	2 (1.4%)	0	0.07
Minor Complications	Pericardial effiusion without requiring intervention	5 (3.4%)	6 (1.4%)	0.162
	Hematoma requiring intervention	3 (2.0%)	4 (1.0%)	0.383
	Hematoma without requiring intervention	7 (4.7%)	15 (3.6%)	0.619
	Vascular complication requiring intervention	3 (2.0%)	3 (0.7%)	0.183
	Displaced electrode portion	2 (1.4%)	2 (0.5%)	0.276
	Blood transfusion	4 (2.7%)	4 (1.0%)	0.214
	Pneumothorax	1 (0.7%)	3 (0.7%)	1
	Pulmonary embolism	1 (0.7%)	1 (0.2%)	0.45
	New severe tricuspid valve insufficiency	5 (3.4%)	3 (0.7%)	0.031
	Arm vein thrombosis	1 (0.7%)	9 (2.1%)	0.465
	Acute kidney failure	6 (4.1%)	2 (0.5%)	0.005
	Anesthesia complications without requiring intervention	0	3 (0.7%)	0.572

*Note:* Numbers with percentages are shown.

### 3.3 Procedure‐Related Mortality

Thirteen patients (3.7%) died within 30 days after TLE. Of these, only five deaths were attributable to the TLE procedure, resulting in a procedure‐related mortality rate of 1.4% (Table 4). An additional six patients died before discharge, and two patients died outside the hospital within 30 days, corresponding to an in‐hospital mortality of 3.1%. Among the five presumably procedure‐related deaths, two were due to stroke, two due to post‐interventional cardiogenic shock, and one due to post‐interventional distal aortic embolic occlusion. Periprocedural outcomes did not differ significantly between groups, while there was a non‐significant trend toward higher 30‐day mortality in patients with right‐sided CIED implantation (5.4% vs. 2.1%, *p* = 0.052).

## 4 Discussion

In this study, we compared baseline characteristics, procedural parameters, and outcomes of TLE according to CIED implantation side. Our results demonstrate that RS lead extraction is associated with distinct patient characteristics, increased procedural complexity, reduced radiological success, and higher complication rates compared with left‐sided extraction, despite similarly high rates of overall clinical success.

### 4.1 Patient Characteristics and Indication for Extraction

Patients with right‐sided CIED implantation were older, more frequently pacemaker‐dependent, and had significantly longer lead dwell times. These findings are consistent with prior reports showing that RS CIED implantation is often chosen in patients with limited venous access, previous device revisions, or anatomical constraints, resulting in a more complex extraction substrate over time [1, 2]. Longer dwell time and pacemaker dependency are well‐established predictors of extraction difficulty and complications [3, 4, 5]. The underlying cardiac disease differed substantially between groups. Right‐sided implantation was predominantly observed in patients with primary electrical heart disease, whereas structural heart disease (including coronary artery disease, dilated cardiomyopathy, and valvular disease) was more common in the left‐sided group. This distribution likely reflects historical implantation strategies (RS CIED was more common during the beginning of CIED implantation) [6] and the frequent need for repeated device revisions in younger patients with inherited arrhythmia syndromes, ultimately leading to right‐sided implantation [6]. During the early years of CIED therapy, right‐sided implantation was more frequently performed and the choice of implantation side was often influenced by operator preference and local practice patterns. As contemporary implantation strategies increasingly favor the left pectoral approach, right‐sided systems encountered in current extraction cohorts are more likely to represent older devices with longer lead dwell times. In addition, patients with inherited arrhythmia syndromes are typically implanted at a younger age and may require multiple device revisions throughout their lifetime, increasing the likelihood of alternative implantation strategies over time.

Indications for extraction also varied between groups: Lead dysfunction was more frequent in left‐sided systems, whereas systemic infection and device upgrade were relatively more common in right‐sided systems. This may reflect the higher number of prior device procedures and longer lead dwell times in the right‐sided group, both of which are known risk factors for device‐related infection and the need for system upgrades [7, 8].

### 4.2 Procedural Complexity

Procedural duration was significantly longer in right‐sided lead extractions, and there was a trend toward increased fluoroscopy time. In addition, femoral snare techniques were used more frequently. These findings suggest increased procedural complexity, likely attributable to unfavorable lead orientation, altered traction vectors, and more extensive fibrotic adhesions in right‐sided systems, as well as increased complexity due to higher dwelling times and a higher presence of passive fixation leads [9, 10]. From a technical perspective, left‐sided extractions benefit from a more direct alignment between the superior vena cava, right atrium, and right ventricle, facilitating coaxial traction. In contrast, right‐sided lead extraction often requires more complex maneuvers to overcome acute angulations and venous curves, potentially explaining the increased need for adjunctive femoral approaches observed in our cohort [11].

### 4.3 Success Rates

Although complete radiological success was slightly lower in the RS group, overall clinical success remained high and comparable between groups. This finding is clinically important, as it supports the concept that small retained lead fragments do not necessarily compromise clinical outcomes, particularly in the absence of ongoing infection [3, 12]. Current guidelines emphasize clinical rather than purely radiological success as the primary procedural endpoint, a position supported by our data [1].

### 4.4 Complications and Safety

Major and minor complications, as well as re‐interventions due to complications, were significantly more frequent in RS lead extractions. This aligns with previous studies identifying lead dwell time, multiple prior procedures, and procedural complexity as key determinants of adverse events during extraction [4, 5, 13]. Whether anatomic differences of TLE in RS‐CIED also influenced the complication rate is impossible to determine, because of the retrospective analysis and significant baseline differences. While periprocedural mortality was low and comparable between groups, we observed a trend toward higher 30‐day mortality in the right‐sided group. Although not statistically significant, this finding may reflect the higher burden of comorbidities, increased procedural complexity, and greater incidence of complications in this population rather than a direct effect of implantation side alone. Larger multicenter studies with prospective evaluation are required to further clarify this association.

### 4.5 Clinical Implications

Our findings suggest that right‐sided lead extraction should be recognized as a higher‐risk procedure and warrants meticulous preprocedural planning, including detailed imaging, consideration of advanced extraction tools, and performance in high‐volume centers with immediate surgical backup. These considerations are in line with current international recommendations for complex lead extraction procedures [1, 14]. Importantly, despite increased procedural complexity and complication rates, high clinical success can be achieved, underscoring the feasibility of right‐sided lead extraction when performed by experienced operators.

### 4.6 Limitations

This study is subject to the inherent limitations of its observational and retrospective design. Residual confounding cannot be excluded, and the decision for right‐sided implantation may itself represent a surrogate marker for procedural complexity rather than an independent risk factor. Prospective, multicenter investigations with standardized protocols are warranted to validate these findings and to refine risk stratification models that consider implantation site among other patient and device factors.

## 5 Conclusions

Right‐sided CIED TLE is associated with older patient age, longer lead dwell times, greater procedural complexity, and higher complication rates compared to LS TLE. Nevertheless, clinical success remains high in both groups. These findings highlight the importance of individualized risk assessment and specialized expertise when planning TLE in patients with RS CIED systems.

## Funding

The authors have nothing to report.

## Ethics Statement

The gathering and analysis of all patient data for this paper adheres to the Swiss Ordinance on Human Research with the Exception of Clinical Trials (Human Research Ordinance) and was approved by the local ethics committee (BASEC‐NR: 2018‐01540). All patient data was anonymized. This study was conducted according to the guidelines for good clinical practice and the Declaration of Helsinki.

## Consent

Written informed consent was acquired in all patients for participation in the study.

## Conflicts of Interest

Dr. Hofer reports educational grants, consultant or speaker fees and fellowship support from Abbott, Medtronic, Biotronik, Boston Scientific, Biosense Webster, Novartis, Bayer, Pfizer, Spectranetics. Dr. Breitenstein has received consultant and/or speaker fees from Abbott, Bayer Healthcare, Biosense Webster, Biotronik, Boston Scientific, Bristol‐Myers Squibb, Cook Medical, Daiichi Sankyo, Medtronic, Pfizer, and Spectranetics/Philips. Prof. Steffel has received consultant and/or speaker fees from Abbott, Amgen, Astra‐Zeneca, Bayer, Berlin‐Chemie, Biosense Webster, Biotronik, Boehringer‐Ingelheim, Boston Scientific, Bristol‐Myers Squibb, Daiichi Sankyo, Medscape, Medtronic, Merck/MSD, Novartis, Portola, Roche Diagnostics, Pfizer, Portola, Saja, Servier, and WebMD. He reports ownership of CorXL. Prof. Steffel has received grant support through his institution from Abbott, Bayer Healthcare, Biosense Webster, Biotronik, Boston Scientific, Daiichi Sankyo, and Medtronic. Dr. Häeberlin has received travel fees/educational grants from Medtronic, Biotronik, Abbott, and Philips/Spectranetics without impact on his personal remuneration. He serves as a proctor for Medtronic. He has received research grants from the Swiss National Science Foundation, the Swiss Innovation agency Innosuisse, the Swiss Heart Foundation, the University of Bern, the University Hospital Bern, the Velux Foundation, the Hasler Foundation, the Swiss Heart Rhythm Foundation, and the Novartis Research Foundation. He is Co‐founder and CEO of Act‐Inno AG. The remaining authors declare no conflicts of interest.

## Data Availability

The data that support the findings of this study are available on request from the corresponding author. The data are not publicly available due to privacy or ethical restrictions. Upon urgent request and associated need, our data are available, while our utmost intention is to protect our patient's privacy.
